# The Frequency of Patient-Initiated Violence and Its Psychological Impact on Physicians in China: A Cross-Sectional Study

**DOI:** 10.1371/journal.pone.0128394

**Published:** 2015-06-01

**Authors:** Jianwei Shi, Sheng Wang, Ping Zhou, Leiyu Shi, Yu Zhang, Fei Bai, Di Xue, Xinkai Zhang

**Affiliations:** 1 Key Lab of Health Technology Assessment, Ministry of Health (Fudan University); Collaborative Innovation Center of Social Risks Governance in Health; School of Public Health, Fudan University, Shanghai, 200032, P.R. China; 2 School of Business, East China Normal University, Shanghai, 200241, P.R. China; 3 Bloomberg School of Public Health, Johns Hopkins University, Baltimore, Maryland, 21205, United States of America; 4 Health and Family Planning Commission of Hubei Province, Hubei, 430079, P.R. China; 5 Health and Family Planning Commission of Gansu Province, Gansu, 730030, P.R. China; 6 Shanghai Mental Health Center, Shanghai, 200030, P.R. China; The Ohio State University, UNITED STATES

## Abstract

**Introduction:**

In China, the severity of medical disputes has greatly increased during the past two decades, which has caused various adverse outcomes for health professionals. Previous research on violence in healthcare settings has primarily examined the occurrence of patient-initiated violence and its effects on physicians, but few studies have focused on the impact of the extent of physicians’ exposure to violence. This study examined the different frequency levels of specific types of violence and their relationships to physicians’ psychological wellbeing, including emotional exhaustion (EE), job satisfaction (JS), and intention to leave (IL).

**Methods:**

Using a stratified random sampling method, the participants were drawn from 123 public hospitals in Shanghai, Hubei Province, and Gansu Province in China, and 1,656 completed questionnaires were collected. Chi-square test, analysis of variance, and mixed linear model were employed in the analysis.

**Results:**

The results showed that the rate of exposure to verbal abuse (VA) was the highest (92.75%), followed by threats of assault (TA, 88.10%) and physical assaults (PA, 81.04%). Physicians’ reported high-frequency exposure to VA, TA, and PA was 35.14%, 27.72%, and 19.32%, respectively. The results indicated that exposure to violence significantly affected EE, JS, and IL, and the intensity of the harm of high-frequency exposure was several times stronger than that of low-frequency exposure. Interestingly, we found that VA produced a greater adverse impact on physicians’ EE and satisfaction with work (JS-1) than did TA or PA. This finding may be attributed to the fact that physicians are more likely to be exposed to a high frequency of VA, and the effect of high-frequency exposure is much stronger.

**Conclusion:**

The results indicated that decreasing violent incidents and creating a safer work environment for physicians should be a top priority for both government and society.

## Introduction

Workplace violence refers to violent acts that are directed toward people at work or on duty [1]. It encompasses various types of violence, including verbal abuse (VA), aggression, harassment, bullying, and physical violence such as physical assaults (PA) and threats of assault (TA) [2,3]. Patient-initiated workplace violence in healthcare settings has become an increasingly serious problem in many countries [4–7]. Health professionals in the UK, especially general practitioners (GPs), are consistently at risk of this type of violence [4]. A study on Australian GPs found that 63.7% of those sampled had experienced violence at work [5]. One survey reported that the prevalence of violence toward physicians in emergency departments in Morocco was 70% [8]. In the US, deteriorating physician-patient relationships have been evident since the 1990s [9–11]. In developing countries, a recent study showed that more than half of the surveyed health personnel experienced at least one incident of violence in 2011 [7]. In China, the severity of medical disputes has been increasing significantly over the past two decades. Due to a lack of trust between physicians and patients and an imperfect legal system that provides weak safeguards of the rights and interests of both healthcare professionals and patients, patient-initiated violence against physicians has been on the rise. Patient-initiated violence has become one of the most important issues in China’s new healthcare system reform [12–13].

Workplace violence or aggression can result in a variety of adverse outcomes for health professionals, including poor psychological status, poor work performance, and negative impact on quality of life [8, 14–19]. Empirical evidence has shown that these adverse outcomes lead to an increase in physician turnover intentions and deteriorating relationships between physicians and patients [7, 20–21]. In ethical reviews of research involving human subjects, specific risks are frequently identified, including the probability of a given harm and its severity and duration [22–24]. This assessment approach can also be applied to studying the harm of patient-initiated violence in medical practices. Previous studies have examined the occurrence of patient-initiated violence and its effects on physicians, and the focus tends to be on specific violent incidents. However, few studies have focused on whether different levels (i.e., low probability versus high probability) and different types of violent events (i.e., VA, TA, or PA) produce different impact.

The aim of this study was to investigate the diverse effects of different levels and types of patient-related violence on physicians in terms of emotional exhaustion, job satisfaction and turnover intention. In this study, frequency of violence is defined as the number of times violence occurs within a work setting during a particular period of time. This measure reflects physicians’ perceptions of their work environment, including both directly experienced and indirectly witnessed violent cases. The hypothesis was that the psychological impact of workplace violence varied depending on the level of exposure (i.e., low level vs. high level) and the forms of violence (i.e., VA, TA, or PA).

## Methods

### Sample and procedures

A cross-sectional survey was conducted using a stratified sampling method in Shanghai, Hubei Province, and Gansu Province in China from June through October 2013. The selection of regions was designed to capture varying levels of socioeconomic status and geographic distributions. We first selected three regions that represented high, middle, and low levels of socioeconomic status that were located in the eastern, central, and western parts of China. Then, we selected three districts or prefecture-level cities/counties representing high, middle, and low levels of socioeconomic status within each province/municipality for a total of nine sampled areas. We randomly selected two tertiary hospitals, three to four secondary hospitals, and six to twelve healthcare community centers/township hospitals in each city/county. In Shanghai, six tertiary general hospitals were selected from the region as a whole (because tertiary general hospitals were distributed very unevenly among the districts). In some counties of Hubei and Gansu provinces, the number of physicians in township hospitals was very small, so we increased the number of sampled township hospitals and decreased the number of surveyed physicians within each township hospital in these counties. Ultimately, the study subjects were recruited from 123 hospitals, including 75 healthcare community centers/township hospitals, 30 secondary hospitals, and 18 tertiary public general hospitals. A total of 2,210 physicians affiliated with these hospitals (with at least one year of work experience at the institution) were invited to participate in this survey, and 1,656 valid questionnaires were collected.

### Data collection and measurement

Anonymous, self-administered questionnaires were distributed and collected by coordinators who had been trained to understand the study’s aims, the questionnaire content, and ethical issues. The questionnaire covered the dependent variables, including emotional exhaustion (EE), job satisfaction (JS), and intention to leave (IL). The independent variables consisted of reported patient-initiated violence, participants’ personal characteristics, and other work-related factors.

#### Hypotheses of relationships between patient-initiated violence and psychological variables

Previous studies have primarily focused on the occurrence of patient-initiated violence and its effects on physicians. However, few studies have focused on whether different levels (i.e., low versus high frequency) and types of violence may produce different effects. We hypothesized that exposure to different frequencies and types of violence would produce different effects. In model typeI (MT-I), we modeled the relationship between the occurrence of three types of patient-initiated violence and physicians’ EE, JS, and IL. It was hypothesized that the relationships between patient-initiated violence and both EE and IL would be positive, whereas the relationship with JS would be negative. In model typeII (MT-II), we modeled the impact of different levels of violence on the psychological variables. Next, it was hypothesized that compared with a low frequency of violence, associations between high frequency of violence and the dependent variables would be considerably enhanced (see Table 1).

**Table 1:** Hypotheses of the relationship between patient-initiated violence and psychological variables.

					Dependent variable					
Model type	Independent variable	Type of variable	Value	Ref.	Emotional exhaustion (EE)	Job satisfaction (JS)		Intention to leave (IL)		
						Satisfaction with work (JS-1)	Satisfaction with career (JS-2)	Leave front-line clinical services(IL-1)	Leave the hospital (IL-2)	Quit practicing medicine (IL-3)
MT-I	Verbal abuse(VA)	binomial	0:none	none	+	-	-	+	+	+
			1: occurrence							
MT-I	Threats of assault(TA)	binomial	0:none	none	+	-	-	+	+	+
			1:occurrence							
MT-I	Physical assault(PA)	binomial	0:none	none	+	-	-	+	+	+
			1:occurrence							
MT-II	Verbal abuse(VA)	ordinal	0:none	none	low freq.:+	low freq.: -	low freq.: -	low freq.:+	low freq.:+	low freq.:+
			1:low freq.		high freq:++	high freq: - -	high freq: - -	high freq:++	high freq:++	high freq:++
			2:high freq.							
MT-II	Threats of assault(TA)	ordinal	0:none	none	low freq.:+	low freq.: -	low freq.: -	low freq.:+	low freq.:+	low freq.:+
			1:low freq.		high freq:++	high freq: - -	high freq: - -	high freq:++	high freq:++	high freq:++
			2:high freq.							
MT-II	Physical assault(PA)	ordinal	0:none	none	low freq.:+	low freq.: -	low freq.: -	low freq.:+	low freq.:+	low freq.:+
			1:low freq.		high freq:++	high freq: - -	high freq: - -	high freq:++	high freq:++	high freq:++
			2:high freq.							

Note. Control variables considered in MT-I and MT-II included personal characteristics and work-related factors (see Table 2);

+: positive, -:negative, ++: increasingly positive, --:increasingly negative.

#### Measurement of emotional exhaustion

In this study, EE was assessed using the Maslach Burnout Inventory [25], which includes the following 4 items: (1) I feel emotionally drained from my work; (2) I feel exhausted at the end of the work day; (3) I feel fatigued when I get up in the morning and have to face another day on the job; and (4) Working all day is really a strain for me. The response scale included the following 7 categories: never, rarely, occasionally, sometimes, often, frequently, and always. The Cronbach’s alpha of EE (0.934) and the split-half reliability (0.917) were acceptable. With regard to validity, the standardized regression weights of each item on this scale were in the range of 0.863 to 0.909, and the average variance extracted (AVE) [26] of this scale was 0.784. Thus, the tests showed that the convergent validity was good.

#### Measurement of job satisfaction

JS was measured using 6 items derived from Konrad et al.’s Global Satisfaction Scales that are specifically for physicians [27], including satisfaction with work (JS-1) and satisfaction with career (JS-2). An example of work satisfaction was: “Overall, I am pleased with my work”. An example of career satisfaction was:“If I were to choose over again, I would still become a physician”. The Cronbach’s alpha coefficients of JS-1 and JS-2 were 0.918 and 0.903, respectively, and the split-half coefficients were 0.898 and 0.870, respectively. Furthermore, the data showed that the convergent validity of the scales was acceptable, with the standardized regression weights of each item for JS-1 (0.834–0.966) and JS-2 (0.784–0.927) in the appropriate range. The AVE for JS-1 and JS-2 was 0.794 and 0.764, respectively. The participants’ responses were recorded on a 6-point Likert scale that ranged from 1 (extremely dissatisfied) to 6 (extremely satisfied).

#### Measurement of intention to leave

In this study, IL was designed based on Williams et al.’s research [28], reflecting three types of intention: intention to leave front-line clinical services within one’s hospital (IL-1), intention to leave one’s hospital (IL-2), and intention to quit practicing medicine (IL-3). Three types of IL were identified: (1) I would most likely change my work from front-line clinical services to back-office support in this hospital within the next 2 years; (2) I would most likely leave this hospital within the next 5 years; and (3) I would most likely quit practicing medicine within the next 5 years. The responses ranged from extremely disagree (= 1) to extremely agree (= 6).

#### Measurement of patient-initiated violence

Following a previous study [29], patient-initiated violence was measured with questions that asked whether the respondent had directly experienced or indirectly witnessed patient-initiated violence in his/her hospital, including the following items: (1) How often have you or your colleagues suffered from VA by patients or their relatives/friends in the past 12 months? (2) How often have you or your colleagues suffered from TA by patients or their relatives/friends in the past 12 months? (3) How often have you or your colleagues suffered from PA from patients or their relatives/friends in the past 12 months? The response categories were: never, rarely [several times (<12 times) in the past 12 months], occasionally (once per month), sometimes (2–3 times per month), often (once per week), frequently (2–5 times per week), and always (almost every day). The occurrence of a specific type of violence was dichotomized as yes (ranging from rarely to always) or no (never). The frequency of patient-initiated violence was categorized into 3 levels: none (never), low (rarely, occasionally, and sometimes), and high (often, frequently, and always).

#### Control variables

The control variables included participants’ personal characteristics (i.e., gender, age, educational level, professional qualifications, years in the profession, annual income, health status, initial vocational motivation, and personal toughness) and work-related factors (i.e., hospital type, work location, department affiliation, night shift, relationships with colleagues and staff, and organizational ethical climate).

The participants’ health status was self-reported on a 5-point scale: very poor, poor, not bad, good, and excellent. Vocational motivation was measured by asking, what was your most important initial reason for becoming a physician: enthusiasm for medicine, solely to make a living, family’s wishes, or other? The personal toughness scale, which was borrowed from Lu and Liang [30], has demonstrated good reliability and validity. In this study, the Cronbach’s alpha coefficient of the personal toughness scale was 0.901. The average of the values of all items was used to reflect personal toughness. The participants’ relationships with colleagues and staff were assessed using the following: how well do I work with my colleagues and with support staff? The responses were recorded on a 6-point scale (“definitely not well” = 1 to “very well” = 6). The organizational ethical climate scale included 5 items that were developed for the current study based on a literature review and consultations with experts. An example is: “For poor patients, my hospital will do its best to treat them and never skimp on treatment or send them away”. The responses were recorded on a 6-point scale (“strongly disagree” = 1 to “strongly agree” = 6). The Cronbach’s alpha coefficient of the organizational ethical climate scale was 0.737. The average of the item values indicated the organizational ethical climate.

### Statistical analysis

All of the data were analyzed using SAS Software 9.20 (SAS Institute Inc., Cary, NC, USA). To analyze the psychological status of the respondents, we calculated the average scores for all of the items used to measure EE, JS-1 and JS-2. Basic descriptive statistics was used to analyze personal characteristics, work-related factors, patient-initiated violence, and the psychological variables. Chi-square test was applied to compare the differences in exposure to various levels of frequency of workplace violence among two or more groups. Analysis of variance (ANOVA) was used to explore discrepancies in personal toughness, relationships with colleagues or staff, and organizational ethical climate among different frequency levels of violence. We also estimated multivariate models of the relationship between various types of patient-initiated violence and EE, JS (JS-1, JS-2), and IL (IL1-IL3), respectively. We used a mixed linear model with the function “PROC MIXED” in SAS 9.20 to allow for correlation across respondents within hospitals. The mixed linear model was conducted in two models mentioned in Table 1. Both models were implemented while controlling for personal characteristic variables and work-related factors. The statistical models were separately tested for VA, TA, and PA.

## Ethics Statement

Research ethics approval for this study was granted by the Institutional Review Board (IRB) of the School of Public Health, Fudan University (ref: 2012-03-0338) with a waiver of documentation of informed consent for two reasons: (1) this study presented minimal risk of harm to its subjects; and (2) the questionnaires were returned anonymously. During the investigation, each potential subject read a written statement about the survey’s purpose and content and the subject’s rights and interests. All of the subjects provided informed consent.

## Results

### Distribution of personal characteristics and work-related factors

The respondents’ demographics and work-related factors by types of public healthcare organization in China are shown in Table 2. Overall, the proportions of respondents who practiced in community health centers/township hospitals, secondary hospitals, and tertiary public hospitals were 24.64%, 37.62%, and 37.74%, respectively. Slightly more than half of the physicians were male (54.05%); 47.04% were 30–39 years old; approximately half or more held bachelor’s degrees (60.51%), were residents and below (47.55%), had 1–10 years of experience in the profession (51.39%), had an annual income of less than 50,000 RMB (50.54%), and reported very poor, poor, or not bad health status (65.52%). “Enthusiasm for medicine” and “solely for a living” were cited as the major initial vocational motivation by 45.71% and 33.88% of the respondents, respectively. The average personal toughness score was 4.85 (SD = 0.85), with physicians who worked in tertiary public general hospitals displaying the lowest scores.

**Table 2:** Basic descriptive analysis of the respondents, n(%).

Variable	Community health center/ township hospital	Secondary public hospital	Tertiary public hospital	Total
	n = 408	n = 623	n = 625	n = 1656
***Personal characteristics***				
Gender				
Male	191(46.81)	366(58.75)	338(54.08)	895(54.05)
Female	217(53.19)	257(41.25)	287(45.92)	761(45.95)
Age (years)				
<30	77(18.87)	106(17.01)	154(24.64)	337(20.35)
30–39	162(39.71)	285(45.75)	332(53.12)	779(47.04)
40–49	106(25.98)	180(28.89)	111(17.76)	397(23.97)
≥50	63(15.44)	52(8.35)	28(4.48)	143(8.64)
Professional qualifications (Missing = 24)				
Resident or below^&^	229(57.39)	269(43.60)	278(45.13)	776(47.55)
Attending physician	125(31.33)	213(34.52)	204(33.12)	542(33.21)
(Associate) chief physician	45(11.28)	135(21.88)	134(21.75)	314(19.24)
Education^$^				
Associate’s degree or below	188(46.08)	74(11.88)	26(4.16)	288(17.39)
Bachelor’s degree	209(51.23)	493(79.13)	300(48.00)	1002(60.51)
Master’s degree or above	11(2.70)	56(8.99)	299(47.84)	366(22.10)
Years in the profession (years)				
1–5	90(22.06)	139(22.31)	217(34.72)	446(26.93)
6–10	73(17.89)	161(25.84)	171(27.36)	405(24.46)
11–15	66(16.18)	81(13.00)	90(14.40)	237(14.31)
16–20	58(14.22)	116(18.62)	86(13.76)	260(15.70)
>20	121(29.66)	126(20.22)	61(9.76)	308(18.60)
Annual income (RMB)				
<50,000	250(61.27)	355(56.98)	232(37.12)	837(50.54)
[50,000–100,000)	123(30.15)	222(35.63)	229(36.64)	574(34.66)
≥100,000	35(8.58)	46(7.38)	164(26.24)	245(14.79)
Health status				
Very poor or poor	45(11.03)	87(13.96)	94(15.04)	226(13.65)
Not bad	184(45.10)	320(51.36)	355(56.80)	859(51.87)
Good or great	179(43.87)	216(34.67)	176(28.16)	571(34.48)
Initial vocational motivation				
Enthusiasm for medicine	212(51.96)	281(45.10)	264(42.24)	757(45.71)
Solely for a living	113(27.70)	228(36.60)	220(35.20)	561(33.88)
Family’s wishes	50(12.25)	72(11.56)	87(13.92)	209(12.62)
Others	33(8.09)	42(6.74)	54(8.64)	129(7.79)
Personal toughness, M(SD)	4.93(0.74)	4.90(0.87)	4.76(0.88)	4.85(0.85)
***Work-related factors***				
Location				
Shanghai	132(32.35)	169(27.13)	235(37.60)	536(32.37)
Gansu	122(29.90)	245(39.33)	195(31.20)	562(33.94)
Hubei	154(37.75)	209(33.55)	195(31.20)	558(33.70)
Department				
Internal medicine	37(9.07)	201(32.26)	170(27.20)	408(24.64)
Surgery	14(3.43)	170(27.29)	150(24.00)	334(20.17)
Gynecology/Obstetrics/Pediatrics	20(4.90)	76(12.20)	105(16.80)	201(12.14)
ENT	10(2.45)	66(10.59)	96(15.36)	172(10.39)
Medical technique	28(6.86)	96(15.41)	83(13.28)	207(12.50)
General medicine and prevention care	153(37.50)	0(0.00)	5(0.80)	158(9.54)
Others	146(35.78)	14(2.25)	16(2.56)	176(10.63)
Night shift				
No	231(56.62)	139(22.31)	111(17.76)	481(29.05)
Yes	177(43.38)	484(77.69)	514(82.24)	1175(70.95)
Relationships with colleagues, M(SD) (Missing = 21)	4.53(1.28)	4.21(1.40)	4.16(1.28)	4.27(1.33)
Relationships with staff, M(SD) ^§^ (Missing = 6)	4.65(1.19)	4.27(1.26)	4.13(1.23)	4.31(1.25)
Organizational ethical climate, M(SD)	4.73(0.73)	4.59(0.90)	4.52(0.80)	4.59(0.83)

Note. &In China, residents and below usually includes residents and physician assistants. "Residents" refer to those who have graduated from medical school and are in clinical training. Usually, they have to operate under the guidance of attending physicians and/or (associate) chief physicians. They are only permitted to practice some basic activities if they possess the corresponding qualifications after some training and they have obtained a national medical practitioner license. “Physician assistants” almost exclusively work in community health centers or township hospitals. They have graduated from medical school with an associate’s degree or below and have professional qualifications that are less than those of residents. In some township hospitals, especially those located in remote or poor villages, because of a lack of qualified physicians, physician assistants are allowed to provide basic healthcare services after they have passed the assistant medical practitioner’s examination.

^$^In China, most physicians in secondary and tertiary hospitals currently hold a bachelor’s degree or a master’s degree or above. However, there are also some older physicians who have only associate’s degrees but who also have a high level of qualifications because of their abundant medical practice experience in secondary or tertiary hospitals. In community health centers and township hospitals, some healthcare practitioners such as residents or physician assistants still have an associate’s degree or below, and some older attending physicians who work in community health centers or township hospitals also have an associate’s degree.

^§^Staff includes nurses, medical technicians, and logistical personnel.

Of the 1,656 participants, 32.37% were from Shanghai and 33.94% were from Gansu Province. These physicians came from a variety of hospital departments. Furthermore, 70.95% worked the night shift. Physicians in higher-level hospitals (i.e., tertiary hospitals) were more likely to report poorer working relationships with colleagues and staff and a poorer organizational ethical climate.

### Percentages of exposure to the three types of patient-initiated violence at different frequency levels

The percentages of exposure to different frequencies of VA, TA, and PA by personal characteristics and work-related factors are displayed in Table 3. Generally, exposure to VA was the most likely (n = 1536, 92.75%), followed by TA (n = 1459, 88.10%) and PA (n = 1342, 81.04%). Physicians were also most likely to report high-frequency exposure to VA (n = 582, 35.14%), followed by TA (n = 459, 27.72%) and PA (n = 320, 19.32%). Male physicians were more likely to experience high exposure to VA (40.67%), TA (33.52%), and then PA (24.58%). In addition, physicians aged 30–39 reported higher exposure to VA (40.31%) and TA (31.19%). There were no differences between physicians with different professional qualifications, except that residents and below were less likely to report a high frequency of TA (24.74%, p = 0.042). Physicians with a master’s degree or above and those who reported poor or very poor health status were more likely to report a high frequency of VA, TA, and PA. Those who had worked for less than 10 years were the highest-risk group, and those with greater than 20 years of experience were the least likely to report violence. With respect to initial vocational motivation, physicians with an enthusiasm for medicine were at the lowest risk, and those who worked solely for a living were at the highest risk. Physicians with lower scores of personal toughness were more likely to report a high frequency of violence.

**Table 3:** Percentages of exposure to the three types of patient-initiated violence at different frequency levels by personal characteristics and work-related factors, %.

Variable	Sample (n)	Verbal abuse (VA)			Threats of assault (TA)			Physical assault (PA)		
		None	Low Freq.	High Freq.	None	Low Freq.	High Freq.	None	Low Freq.	High Freq.
		(n = 120)	(n = 954)	(n = 582)	(n = 197)	(n = 1000)	(n = 459)	(n = 314)	(n = 1022)	(n = 320)
***Personal characteristics***										
Gender										
Male	895	6.59	52.74	40.67	11.84	54.64	33.52	18.32	57.09	24.58
Female	761	8.02	63.34	28.65	11.96	67.15	20.89	19.71	67.15	13.14
*P-value*			<0.001			<0.001			<0.001	
Age (years)										
<30	337	8.01	61.13	30.86	12.17	63.50	24.33	14.84	66.77	18.40
30–39	779	4.88	54.81	40.31	8.99	59.82	31.19	15.79	63.16	21.05
40–49	397	9.32	61.71	28.97	13.60	62.72	23.68	24.69	59.70	15.62
≥50	143	12.59	53.15	34.27	22.38	49.65	27.97	30.07	47.55	22.38
*P-value*			<0.001			<0.001			<0.001	
Education										
Associate’s degree or below	288	14.93	62.50	22.57	24.65	60.07	15.28	35.07	54.86	10.07
Bachelor’s degree	1002	6.69	56.59	36.73	10.78	60.98	28.24	18.36	61.98	19.66
Master’s degree or above	366	2.73	56.56	40.71	4.92	59.02	36.07	7.92	66.39	25.68
*P-value*			<0.001			<0.001			<0.001	
Professional qualifications										
Resident or below	776	7.73	59.54	32.73	12.63	62.63	24.74	18.17	64.56	17.27
Attending physician	542	6.09	56.46	37.45	9.78	60.33	29.89	19.37	61.07	19.56
(Associate) chief physician	314	7.01	55.10	37.90	11.78	55.73	32.48	18.47	57.01	24.52
*P-value*			0.292			0.042			0.076	
Years in the profession (years)										
1–5	446	6.28	60.09	33.63	10.31	63.68	26.01	13.90	67.04	19.06
6–10	405	6.42	54.32	39.26	9.88	60.00	30.12	14.81	64.69	20.49
11–15	237	5.06	54.85	40.08	9.70	59.49	30.80	17.30	60.76	21.94
16–20	260	6.15	58.46	35.38	10.00	60.77	29.23	19.62	61.15	19.23
>20	308	12.34	59.74	27.92	20.13	56.49	23.38	32.47	51.30	16.23
*P-value*			0.002			<0.001			<0.001	
Annual income (RMB)										
<50,000	837	8.96	58.30	32.74	14.22	61.53	24.25	20.67	62.37	16.97
[50,000–100,000)	574	6.27	56.97	36.76	10.98	58.71	30.31	19.51	60.10	20.38
≥100,000	245	3.67	56.73	39.59	6.12	60.41	33.47	11.84	63.27	24.90
*P-value*			0.019			<0.001			0.005	
Health status										
Very poor or poor	226	3.98	46.46	49.56	5.75	55.75	38.50	12.83	61.95	25.22
Not bad	859	4.77	56.00	39.23	9.08	59.25	31.66	15.13	62.75	22.12
Good or great	571	12.26	64.45	23.29	18.56	63.92	17.51	27.15	60.07	12.78
*P-value*			<0.001			<0.001			<0.001	
Initial vocational motivation										
Enthusiasm for medicine	757	10.70	61.43	27.87	15.59	62.09	22.32	22.19	62.09	15.72
Solely to make a living	561	3.92	50.80	45.28	7.13	57.40	35.47	14.44	61.50	24.06
Family’s wishes	209	4.78	62.20	33.01	10.05	65.07	24.88	18.18	63.64	18.18
Others	129	5.43	57.36	37.21	13.95	55.81	30.23	20.93	57.36	21.71
*P-value*			<0.001			<0.001			0.001	
Personal toughness, M(SD)	1656	5.21(0.70)	4.91(0.81)	4.69(0.90)	5.15(0.71)	4.89(0.81)	4.65(0.92)	5.08(0.74)	4.85(0.84)	4.63(0.90)
*P-value*			<0.001			<0.001			<0.001	
***Work-related factors***										
Hospital type										
Community	408	11.76	61.76	26.47	19.36	63.24	17.40	30.64	57.84	11.52
Secondary	623	8.35	59.23	32.42	12.52	62.28	25.20	19.10	63.40	17.50
Tertiary	625	3.20	53.28	43.52	6.40	56.64	36.96	11.20	62.56	26.24
*P-value*			<0.001			<0.001			<0.001	
Location										
Shanghai	536	4.29	54.66	41.04	7.84	59.70	32.46	16.60	60.07	23.32
Gansu	562	11.92	60.85	27.22	19.04	61.39	19.57	25.98	60.85	13.17
Hubei	558	5.38	57.17	37.46	8.60	60.04	31.36	14.16	64.16	21.68
*P-value*			<0.001			<0.001			<0.001	
Department										
Internal medicine	408	5.39	55.64	38.97	9.31	60.78	29.90	13.97	63.73	22.30
Surgery	334	5.69	50.30	44.01	7.19	54.49	38.32	12.87	57.78	29.34
Gynecology/Obstetrics/Pediatrics	201	5.97	59.20	34.83	10.95	61.69	27.36	17.91	65.17	16.92
ENT	172	4.65	71.51	23.84	11.63	67.44	20.93	20.93	65.70	13.37
Medical technique	207	9.18	55.56	35.27	16.91	57.00	26.09	26.09	61.35	12.56
General medicine and preventive care	158	13.92	57.59	28.48	20.25	61.39	18.35	28.48	58.86	12.66
Others	176	10.23	63.07	26.70	14.77	65.34	19.89	24.43	59.66	15.91
*P-value*			<0.001			<0.001			<0.001	
Night shift										
No	481	9.56	61.54	28.90	18.50	59.67	21.83	28.27	55.72	16.01
Yes	1175	6.30	56.00	37.70	9.19	60.68	30.13	15.15	64.17	20.68
*P-value*			<0.001			<0.001			<0.001	
Relationships with colleagues, M(SD)	1635	4.93(1.29)	4.36(1.30)	3.99(1.33)	4.78(1.31)	4.32(1.29)	3.94(1.35)	4.76(1.30)	4.22(1.30)	3.94(1.35)
*P-value*			<0.001			<0.001			<0.001	
Relationships with staff, M(SD)	1650	4.83(1.31)	4.44(1.17)	3.98(1.28)	4.80(1.18)	4.38(1.20)	3.94(1.28)	4.78(1.17)	4.28(1.21)	3.94(1.29)
*P-value*			<0.001			<0.001			<0.001	
Organizational ethical climate, M(SD)	1656	4.84(0.70)	4.64(0.81)	4.47(0.87)	4.77(0.78)	4.65(0.81)	4.39(0.85)	4.77(0.86)	4.62(0.81)	4.35(0.83)
*P-value*			<0.001			<0.001			<0.001	

Exposure to a high frequency of violence was more likely to occur in higher-level hospitals. Physicians in the departments of internal medicine, surgery, and gynecology/obstetrics/pediatrics were exposed to a higher frequency of violence than those in other departments. Physicians in surgery departments were most likely to report a high frequency of VA, TA, and PA (44.01%, 38.32%, and 29.34%, respectively). A greater proportion of physicians who worked night shifts reported a high frequency of violence. In addition, physicians who were exposed to a high frequency of violence were more likely to work in hospitals with lower average scores for organizational ethical climate and had poorer relationships with their colleagues and staff.

### Mixed linear model analysis

Table 4 describes the associations between types of violence and physicians’ EE, JS, and IL. The fixed effects of the mixed linear model analysis clearly demonstrated that violence had significant adverse effects on EE, JS, and IL. Interestingly, of the three types of violence, exposure to VA caused the largest adverse impact on EE (β = 0.811, p<0.001) and satisfaction with work (JS-1) (β = -0.255, p<0.01), and the coefficients of VA were the largest in MT-I. With regard to career satisfaction (JS-2), only PA produced a significant effect (β = -0.274, p<0.001). With regard to IL, the results differed among the three types of IL. MT-I shows that both VA and TA increased physicians' intentions to leave front-line clinical services (β_VA_ = 0.244, p< 0.05; β_TA_ = 0.270, p<0.01), but these types of violence exerted no significant impact on physicians’ intention to leave the hospital or intention to quit practicing medicine. In addition, among the three types of IL, only physicians' intention to quit practicing medicine was affected by PA (β = 0.198, p<0.05).

**Table 4:** Mixed linear model for the associations between patient-initiated violence and EE, JS, and IL.

MT-I (Ref none)						MT-II (Ref none)				
Dependent variable	Independent variable	β	T value	χ2 likelihood^Δ^	UN(1,1)^#^_subject = hospital code_	Independent variable	β	T value	χ2 likelihood^Δ^	UN(1,1)^#^_subject = hospital code_
Emotional exhaustion (EE)	VA(occurrence)	0.811	6.260***	61.760***	0.221***	VA(Low freq.)	0.520	4.320***	66.600***	0.223***
						VA(High freq.)	1.631	12.700***		
	TA(occurrence)	0.670	6.400***	64.690***	0.226***	TA(Low freq.)	0.424	4.340***	74.840***	0.245***
						TA(High freq.)	1.573	14.180***		
	PA(occurrence)	0.599	6.990***	61.900***	0.210***	PA(Low freq.)	0.386	4.750***	72.040***	0.228***
						PA(High freq.)	1.599	15.470***		
Job satisfaction (JS)										
Satisfaction with work(JS-1)	VA(occurrence)	-0.255	-2.640**	26.770***	0.058**	VA(Low freq.)	-0.171	-1.770	28.220***	0.058***
						VA(High freq.)	-0.494	-4.790***		
	TA(occurrence)	-0.180	-2.300*	28.790***	0.060***	TA(Low freq.)	-0.128	-1.620	29.570***	0.060***
						TA(High freq.)	-0.369	-4.110***		
	PA(occurrence)	-0.170	-2.630**	29.130***	0.061***	PA(Low freq.)	-0.143	-2.190*	29.270***	0.060***
						PA(High freq.)	-0.295	-3.540***		
Satisfaction with career(JS-2)	VA(occurrence)	-0.224	-1.880	40.570***	0.121***	VA(Low freq.)	-0.112	-0.940	46.420***	0.129***
						VA(High freq.)	-0.535	-4.210***		
	TA(occurrence)	-0.189	-1.950	40.970***	0.121***	TA(Low freq.)	-0.130	-1.330	43.860***	0.127***
						TA(High freq.)	-0.402	-3.630***		
	PA(occurrence)	-0.274	-3.460***	40.150***	0.120***	PA(Low freq.)	-0.259	-3.220**	40.800***	0.121***
						PA(High freq.)	-0.344	-3.350***		
Intention to leave (IL)										
Leave front-line clinical services(IL-1)	VA(occurrence)	0.244	1.980*	34.860***	0.104***	VA(Low freq.)	0.158	1.280	31.820***	0.100***
						VA(High freq.)	0.488	3.690***		
	TA(occurrence)	0.270	2.700**	35.570***	0.104***	TA(Low freq.)	0.178	1.780	32.090***	0.101***
						TA(High freq.)	0.607	5.340***		
	PA(occurrence)	0.136	1.650	34.480***	0.103***	PA(Low freq.)	0.026	0.320	24.870***	0.088**
						PA(High freq.)	0.653	6.250***		
Leave the hospital (IL-2)	VA(occurrence)	0.109	0.850	24.630***	0.113***	VA(Low freq.)	-0.004	-0.030	19.240***	0.094**
						VA(High freq.)	0.441	3.230**		
	TA(occurrence)	0.193	1.860	24.640***	0.111***	TA(Low freq.)	0.086	0.830	19.680***	0.092**
						TA(High freq.)	0.594	5.050***		
	PA(occurrence)	0.140	1.640	24.700***	0.112**	PA(Low freq.)	0.027	0.310	16.980***	0.084**
						PA(High freq.)	0.687	6.340***		
Quit practicing medicine(IL-3)	VA(occurrence)	0.165	1.320	22.440***	0.079**	VA(Low freq.)	0.065	0.520	21.270***	0.080**
						VA(High freq.)	0.452	3.370***		
	TA(occurrence)	0.184	1.820	23.070***	0.080**	TA(Low freq.)	0.080	0.790	21.900***	0.081**
						TA(High freq.)	0.565	4.900***		
	PA(occurrence)	0.198	2.360*	22.690***	0.080**	PA(Low freq.)	0.091	1.090	19.760***	0.077**
						PA(High freq.)	0.700	6.590***		

Note. Only the predictors of VA, TA, and PA are presented here. Control variables considered in MT-I and MT-II included personal characteristics and work-related factors (see Table 2);

^Δ^Null Model Likelihood Ratio Test;

^#^ Unstructured variances of the intercepts;

* p<0.05,

** p<0.01,

*** p<0.001.

However, when physicians were separated into low-frequency and high-frequency groups, the results changed (as shown in MT-II). The intensity of harm resulting from high-frequency exposure was several times stronger than that of low-frequency exposure in relation to EE. With regard to JS-1, compared with MT-I, MT-II showed that only high-frequency exposure to VA (β = -0.494, p<0.001) and TA (β = -0.369, p<0.001) significantly influenced this variable. With regard to JS-2, MT-II showed that exposure to a high frequency of VA and TA also generated a considerably adverse impact. Both low and high frequencies of PA had remarkable effects on two types of JS. With respect to IL, for all three types of intention to leave, low-frequency exposure to VA, TA, or PA had little impact, whereas high-frequency exposure was related to significantly greater IL. Exposure to a high frequency of PA had the greatest impact on intention to leave front-line clinical services (β = 0.653, p<0.001), intention to leave the hospital (β = 0.687, p<0.001), and intention to quit practicing medicine (β = 0.700, p<0.001). Additionally, exposure to a high frequency of VA (β = 0.488, p<0.001) or TA (β = 0.607, p<0.001) exerted the largest impact on intention to leave front-line clinical services, whereas exposure to a high frequency of PA (β = 0.700, p<0.001) had the greatest influence on physicians' intentions to quit practicing medicine.

In the mixed linear model analysis, all of the models were calculated while controlling for the personal characteristics variables and work-related factors.

## Discussion

The results showed that more than 90% of the respondents were exposed to at least one type of violence in the 12 months prior to the survey. The occurrence rates of VA, TA, and PA were all considerably high (92.75%, 88.10%, and 81.04%, respectively). Moreover, the proportions of physicians who reported exposure to a high frequency of VA, TA, and PA were 35.14%, 27.72%, and 19.32%, respectively. Of the violence types, the risk of VA was the most serious. The deterioration of the relationship between physicians and patients in China has been noted [13,31–32]. In this study, physicians’ exposure to violence was significantly related to their higher EE, lower JS, and higher IL. Notably, we found that the adverse impact of high-frequency exposure to violence on physicians was greater than that of low-frequency exposure. Effective measures must be taken to end violence against physicians in China.

### The potential predictors of exposure to patient-initiated violence

Previous studies have indicated several potential predictors of exposure to patient-initiated violence, such as gender, age, educational level, years in the profession, and department [5,18,33], but their conclusions have not been consistent. One study that used Australian urban data reported that violence was more likely toward female GPs and that more experienced GPs encountered less violence [5]. A study that used data from Fujian and Henan Provinces in China showed that male medical professionals, those who were middle aged (30–45 years), and those with a bachelor’s degree experienced a higher incidence of violence [18]. A study of GPs in the UK found that sex was only a predictor of sexual harassment and that number of years in the profession was a predictor only of intimidation [33]. In this study, male physicians, those with a master’s degree or above and those who had 1–10 years of experience in the profession were more likely to experience a high frequency of violence. These differences may be partially due to cultural factors, methodological differences such as the definitions of various types of violence, or sampling techniques.

However, we posit that female physicians may have better communication skills and, thus, may be better able to manage some potentially problematic situations. Physicians with more years in the profession may be more experienced in medical and social skills. It seems counterintuitive that physicians with a higher educational level are more likely to be exposed to a high frequency of violence. In China, most young physicians have a master’s degree or higher, especially in tertiary hospitals. Patients typically assume that physicians with a higher educational degree should have better medical capabilities and skills. If the outcomes do not meet a patient’s expectations, disputes may occur.

Additionally, physicians with poorer health status, lower personal toughness, who worked in tertiary hospitals located in Shanghai or Hubei Province, who specialized in surgery, and who worked the night shift were more likely to be exposed to a high frequency of violence. Tertiary hospitals are more likely to treat patients with more difficult diseases. Medical accidents that occur in surgical departments may produce more significant damage to patients and their families than those that occur in other departments; thus, conflicts may be more likely to occur, especially TA or PA. It has been reported that violence is more likely to occur between 4 p.m. and 8 a.m. [34]. Other researchers have supported this finding [1, 20]. The higher rates of violence during the evening and night hours may be due to the types and conditions of patients who seek treatment during later hours, such as intoxicated patients and those with violence-related injuries [35].

### More attention to physicians’ exposure to a high frequency of violence

Previous studies have explored the associations between workplace violence and healthcare professionals’ psychological status, such as job stress, anxiety, and job satisfaction [21,36–40]. However, it was not clear from these studies whether the intensity of the adverse effects changed with the frequency of violence. The current study found that exposure to VA, TA, or PA was significantly related to higher EE and lower JS-1, but the intensity of these adverse effects multiplied for those in the high-exposure group. Consequently, low-frequency exposure to VA or TA does not significantly decrease JS-2, but high-frequency exposure notably decreases JS-2. Furthermore, high-frequency exposure to VA, TA, or PA was related to greater IL. The effect of high-frequency exposure to PA on IL was much greater than that of exposure to VA and TA. This result is similar to that of another study that found that bullying is a significant determinant in predicting intention to leave [39].

People generally believe that PA is more dangerous than VA. As a result, the adverse effects of VA are often ignored. The current study showed that VA produced a greater adverse impact on physicians’ psychological status than did TA or PA for EE and satisfaction with work (JS-1). We suspect that physicians are more likely to be exposed to a high frequency of VA, and the effect of high-frequency exposure is much stronger. Physicians may be able to tolerate low-frequency exposure to violence. However, an increase in the frequency of violence can generate a qualitative change; for example, the rate of turnover intention increases significantly, and more physicians feel disappointed with their careers. This result is supported by a study’s [31] findings that more physicians have abandoned medicine and that significantly fewer physicians want their children to work in the medical profession in China than in the past.

### Strengths and limitations

The major strengths of this study include its reasonably large sample size and the empirical finding that high-frequency exposure to VA, TA, and PA has a greater effect on physicians’ EE, JS, and IL than does low-frequency exposure. Furthermore, VA has a greater effect than the other types of violence in the healthcare setting. However, this study also has various limitations. First, the data were based on self-reports, which may involve recall/report bias. Second, causal associations between violence and the physicians’ psychological variables cannot be established in this study because of its cross-sectional nature. It will be necessary to conduct a prospective study to identify causal relationships. Third, the duration of exposure to violence was not collected in this study because it was difficult for the respondents to recall the timing of such events in a retrospective survey. Furthermore, it is worth noting that the measurement of patient-initiated violence in our study covered both experienced and witnessed cases because we intended to explore the effect of such exposure in a work environment with a low or high frequency of violence. Thus, the rate of occurrence of violence that we measured was for a work setting and not for individual physicians. Therefore, the reported occurrence of violence was relatively high. Finally, although our sample size was reasonably large, the results may not be generalizable to the whole nation because the study took place in only three regions of a very large country.

## Conclusion

The results reveal a serious problem in China’s healthcare system and indicate that reducing the frequency of violent incidents and creating a safer work environment for physicians should be a top priority for both government and society. All effective measures should be taken without delay. Hospital administrators, healthcare policy makers, and society should pay more attention to physicians’ high exposure to violence.
